# Early-Life Stress Is Associated with Gender-Based Vulnerability to Epileptogenesis in Rat Pups

**DOI:** 10.1371/journal.pone.0042622

**Published:** 2012-08-03

**Authors:** Sébastien Desgent, Sandra Duss, Nathalie T. Sanon, Pablo Lema, Maxime Lévesque, David Hébert, Rose-Marie Rébillard, Karine Bibeau, Michèle Brochu, Lionel Carmant

**Affiliations:** 1 Centre de Recherche du Centre Hospitalier Universitaire Sainte-Justine, Université de Montréal, Montréal, Québec, Canada; 2 Département de Physiologie, Faculté de Médecine, Université de Montréal, Montréal, Québec, Canada; Hôpital Robert Debré, France

## Abstract

During development, the risk of developing mesial temporal lobe epilepsy (MTLE) increases when the developing brain is exposed to more than one insult in early life. Early life insults include abnormalities of cortical development, hypoxic-ischemic injury and prolonged febrile seizures. To study epileptogenesis, we have developed a two-hit model of MTLE characterized by two early-life insults: a freeze lesion-induced cortical malformation at post-natal day 1 (P1), and a prolonged hyperthermic seizure (HS) at P10. As early life stressors lead to sexual dimorphism in both acute response and long-term outcome, we hypothesized that our model could lead to gender-based differences in acute stress response and long-term risk of developing MTLE. Male and female pups underwent a freeze-lesion induced cortical microgyrus at P1 and were exposed to HS at P10. Animals were monitored by video-EEG from P90 to P120. Pre and post-procedure plasma corticosterone levels were used to measure stress response at P1 and P10. To confirm the role of sex steroids, androgenized female pups received daily testosterone injections to the mother pre-natally and post-natally for nine days while undergoing both insults. We demonstrated that after both insults females did not develop MTLE while all males did. This correlated with a rise in corticosterone levels at P1 following the lesion in males only. Interestingly, all androgenized females showed a similar rise in corticosterone at P1, and also developed MTLE. Moreover, we found that the cortical lesion significantly decreased the latency to generalized convulsion during hyperthermia at P10 in both genders. The cortical dysplasia volumes at adulthood were also similar between male and female individuals. Our data demonstrate sexual dimorphism in long-term vulnerability to develop epilepsy in the lesion + hyperthermia animal model of MTLE and suggest that the response to early-life stress at P1 contributes significantly to epileptogenesis in a gender-specific manner.

## Introduction

Mesial temporal lobe epilepsy (MTLE) is the most prevalent form of refractory epilepsy in humans and is characterized by seizures originating in limbic structures [1]. Although MTLE typically begins in teenage years or even adulthood, the initial insult is thought to occur in early life typically as prolonged febrile seizures. Recent evidence suggests that prolonged febrile seizures themselves could only represent the second insult, occurring mostly in individuals with anatomic or genetic predisposing factors [2].

To study the physiology and biological substrates of the dual pathology in MTLE, we have developed a two-hit model of MTLE [3], [4], [5], [6]. The rationale of the model is that the presence of a cortical malformation induced at P1, leads to enhanced susceptibility to a second insult, the hyperthermic seizure (HS) at P10. Indeed, control pups not exposed to the initial insult only experience brief HS and do not develop spontaneous recurrent seizures (SRS), while all lesioned pups exposed to hyperthermia for a similar period of time develop febrile status epilepticus (LHS pups) followed by MTLE after a latent period of approximately 80 days [2], [4], [5], [7]. In comparison, other models of prolonged HS in naïve animals produced by prolonged exposure to high temperature lead to chronic epilepsy in about a third of pups [8], [9], while the rats exposed to the freeze lesion alone do not experience SRS [4].

Since MTLE is thought to be a multi-stage process commencing in early life, we hypothesized that gender-based differences could be observed and that sexual as well as stress hormone influences could be relevant in its pathogenesis. Clinical data support the existence of gender-based differences in patients with MTLE. For example, male patients with MTLE usually have more secondarily generalized tonic-clonic seizures and suffer more seizure-induced damage than females [10], [11]. Selective vulnerability to early life insults has been shown to be linked to the stress response and to demonstrate sexual dimorphism [12]. For instance, consequences of perinatal hypoxia have been well described to express sexual dimorphism, but little is known about gender-based differences in the outcome of prolonged febrile seizures [13]. Sexual dimorphism has also been described in the pathological consequences of the freeze-lesion model [14], but its impact on epileptogenesis has not been assessed because SRS are not observed in the lesion only animals. Only few animal models of MTLE implying early-life stress paradigms exist in the present literature and even fewer have highlighted the importance of gender differences on seizure outcomes [12], [15], [16], [17], [18], [19]. Therefore, the goals of this study were first to determine if gender differences are observed in epileptogenesis in our dual pathology model of MTLE, and second to demonstrate that differences in early-life stress response could underlie gender-based variability.

## Materials and Methods

### Animals and protocol

Twenty pregnant Sprague-Dawley female rats were obtained from Charles River laboratories (St. Constant, QC, Canada) on embryonic day 10 (E10). Pregnant female rats were first divided in two groups. Group 1 was used to study differences in gender-based vulnerability to epileptogenesis following prolonged HS. Group 2 was used to determine if differences in gender-based vulnerability could be reversible by androgenization of females during the perinatal period. In this second group, pregnant dams were injected subcutaneously (s.c.) daily with 2 mg of testosterone dissolved in a peanut oil vehicle (0.1 ml) from E16 until birth of the litter [14]. Each group (males and females) was formed by randomly pooling pups from the different litters that were treated as follows. One day after birth (P1), animals were randomly assigned to receive a freeze-lesion to the right hemisphere in the sensory motor cortex or to a no surgery condition. In group 2, testosterone was sustained by daily injections of the hormone (s.c. 0.05 ml, 2 mg/ml, 1 ml syringe with 30 ½ G needle) for female pups starting at P1 and ending at P9. All injections were performed at 1 PM during the light phase of the light/dark cycle. At P10, pups were arbitrarily assigned to undergo HS according to a method previously described in detail [3], [4], [5], [8]. At P90–120, some rats (n = 32) were implanted with bipolar electrodes for stereotaxic video-electroencephalogram (video-EEG) and then sacrificed for histological examination (see **Table 1** for study design summary). Rat pups were kept with their mother until P23 and then weaned in a 12 hour light/dark cycle with free ad libitum access to food and water. The dam and pups stayed undisturbed in the animal facility except for the periods of cage maintenance (once a week, less than 10 minutes). All procedures for the use and care of animals conformed to policy and guidelines of the Canadian Council for Animal Care (CCAC), and the protocols were approved by institutional rules from the Comité Institutionel des Bonnes Pratiques Animales en Recherche (CIBPAR) at the CHU Ste-Justine Research Center, Université de Montréal.

**Table 1:** Schematic table of study design, experimental groups and investigations.

	Perinatal Testoterone (+T)	Freeze Lesion (L)	Hyperthermia Seizures (HS)	Plasma Corticosterone (2 hours before and after)		Latencies Generalized Convulsion (GC)	Stereotaxic Video EEG	Stereology Cavalieri Volumes
	from E16 to P9	at P1	at P10	L at P1	HS at P10	HS at P10	P90 to P120	P120
**GROUP 1**								
**N**	No	No	No	n = 10M/13F	nil	nil	n = 4M	nil
**L**	No	Yes	No	n = 16M/13F	nil	nil	n = 4M	nil
**HS**	No	No	Yes	nil	n = 10M/10F	n = 15M/14F	n = 4M	nil
**LHS**	No	Yes	Yes	nil	n = 9M/10F	n = 14M/15F	n = 8M/8F	n = 5M/5F
**GROUP 2**								
**L+T**	Yes	Yes	No	n = 13F	nil	nil	nil	nil
**LHS+T**	Yes	Yes	Yes	nil	n = 10F	n = 16F	n = 4F	nil

**M**, Male; **F**, Female; **N**, naïve rats separated from the dam for a period equivalent to the surgery time in the L group at P1; **L**, animals simply subjected to cortical freeze lesion at P1; **HS**, only exposed to hyperthermia-induced febrile seizures at P10; **LHS**, submitted to the combination of cortical freeze lesion at P1 and hyperthermia seizures at P10; **L+T**, subjected to perinatal testosterone administration (from E16 to P9) and cortical freeze lesion at P1; **LHS+T**, perinatal testosterone treatment plus dual postnatal insults at P1 and P10.

### P1 freeze-lesion induction

**Figure 1:**
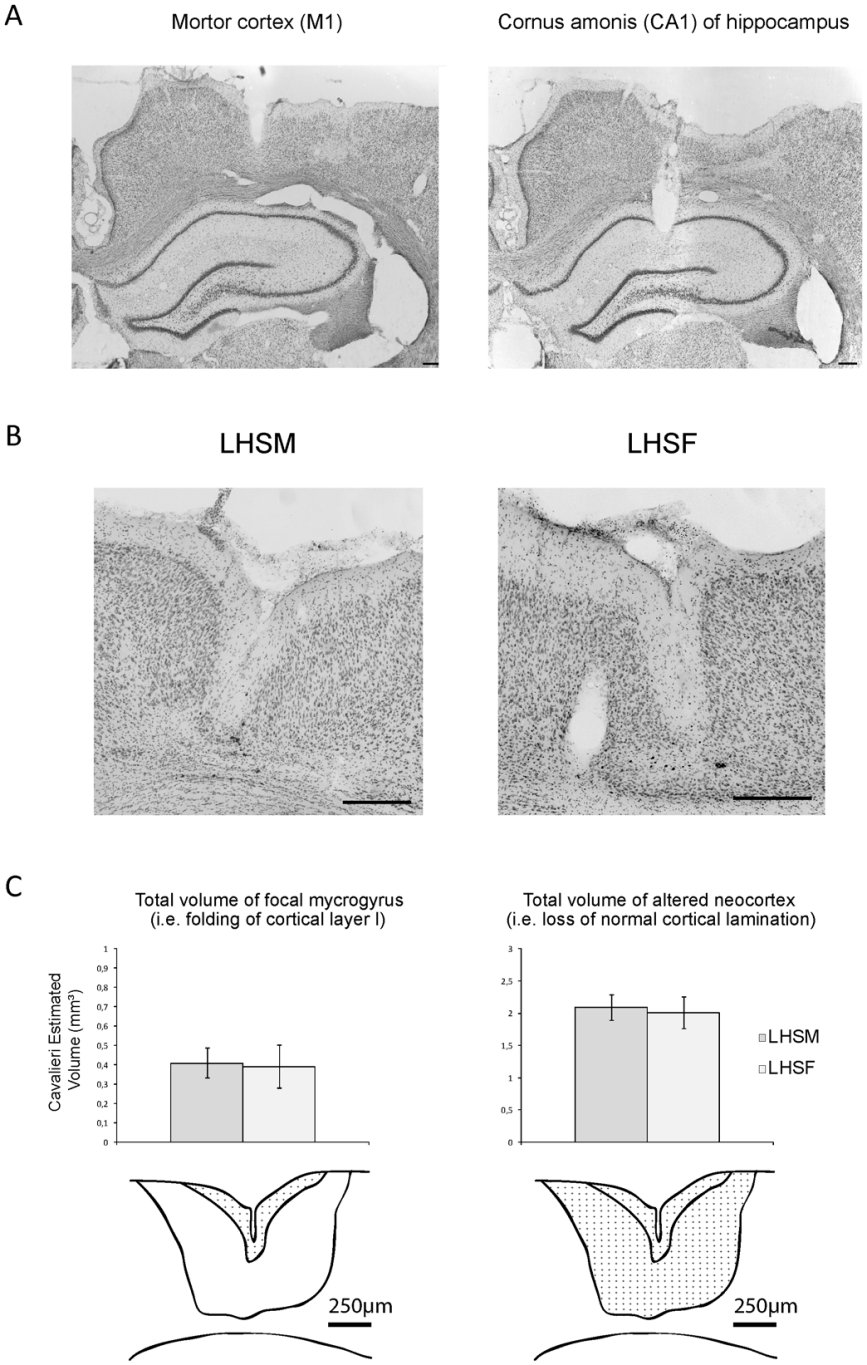
Photomicrographs of cresyl violet sections and Cavalieri's cortical lesion volumes estimations in the adult rats. In **A**), a confirmation example of the bipolar electrodes placement for EEG recordings in a LHSF+T rat is shown in motor cortex M1 (left panel) and in the *cornus ammonis* region one (CA1) of the hippocampus (right panel). **B**) Cresyl violet stained coronal section through the dysplasic lesion in sensorimotor cortex of P120 rat brains, male (left) and female (right), that received a freeze-induced lesion at P1 and hyperthermic seizure at P10 (LHS). Photomicrographs are showing a similar and well-formed, four-layered microgyrus in the middle of each panel for both genders. In **C**), (Top panel) histograms of the volume estimations in the LHSM versus LHSF groups using the Cavalieri's principle for the focal mycrogyrus (left) and total amount of altered neocortex (right). (Bottom panel) diagrams illustrating the region of interest for sampling in each case highlighted with grid points. All Scale bars = 250 µm.

At P1, all manipulations started at 1PM. The dam was removed from the cage and her pups were quickly weighed, separated by gender and assigned equally to one of the experimental groups. This took less than 5 minutes per litter. Immediately, each pup was removed from the box and placed in our anaesthesia induction chamber (oxygen 0.5 L/min and isoflurane 4%) where it remained during 120 seconds. We then positioned the animal in a stereotaxic frame to induce the focal four-layered microgyrus based on a previously detailed technique [5], [14], [20] (**Figure 1**). Briefly, a rostro-caudal incision of the scalp was performed to expose the surface of the skull. A copper probe cooled in liquid nitrogen was then put in contact with the skull overlying the right fronto-parietal cortex for 10 seconds (2 mm caudal to bregma and 2 mm lateral to sagittal suture). The wound was closed with simple suture points. The surgical procedure took less than 7 minutes for each pup. The pups recovered on a thermal pad for 10 minutes and were then returned to the dam after a maximal total separation period of 20 minutes.

### P10 Hyperthermia-induced seizure

In all litters when pups reached postnatal day 10 (P10), they were exposed to HS as previously described [3], [4], [5], [8]. Each pup was individually placed in a plexiglass box in which a hair-dryer produced an indirectly oriented hot air flow to increase their core temperature to 40–43°C. Each pup remained in the box until the onset of generalized convulsion (GC) with the loss of rearing reflex was observed (total mean latency of 7.93±1.46 minutes). Pups were then immediately removed from the box for further behavioral observation. Once pups recovered their righting reflex, they were returned to the dam.

### EEG monitoring

To evaluate the presence of SRS, freely-moving animals from the different experimental groups (see **Table 1**) were implanted at P90 with bipolar electrodes and placed in individual plexiglass cages surrounded by a Faraday tent to undergo video-EEG recordings. A stainless steel bipolar electrode of 200 µm in diameter (Plastics-1 Inc., Roanoke, VA, USA) was positioned into the right motor cortex (M1) and in the dorsal hippocampus (*cornu ammonis* region one, CA1), at the following coordinates with reference to bregma: AP = −3.0, ML = −2.0, DV = 1.0 and AP = −3.7, ML = −2,4, DV = 2.5 respectively (**Figure 1 A**) [21]. EEGs and animal behaviors were recorded simultaneously with a Stellate Harmonie system linked to a 32-channels Lamont amplifying unit and an infrared video camera positioned 1.5 meter in front of the cages (Stellate Systems v 6.2e, Victoria, Montreal, Qc, CAN). Rats were monitored 4 hours a day for 30 days (2 hours during the daytime, from noon to 2 PM, and 2 hours at night, from midnight to 2 AM). Three separate observers blinded to the treatment groups, reviewed the video-EEGs to detect spontaneous recurrent seizures and clinically associated behaviours. Electrographic seizures were defined by the occurrence of episodes of high voltage synchronous spike and/or poly-spike activities. The minimal time period of synchronous activity required for the event to be declared as an electrographic seizure was 5 seconds. Furthermore, typical epileptic behaviors had to be present, in the same period, to diagnose SRS.

### Post EEG histology

After EEG recordings, animals were sacrificed by intraperitoneal (i.p.) injection with a drug mixture of Ketamine (90 mg/kg), Xylazine (10 mg/kg) and Acepomazine (5 mg/kg) (CDMV Inc., Saint-Hyacinthe, QC, Canada), and perfused through the heart with 0.1 M phosphate-buffered saline (PBS; pH 7.4) followed by a phosphate-buffered solution (PB) of 4% paraformaldehyde. Brains were blocked stereotaxically within the skull, removed, post-fixed for two hours at 4°C in a 4% paraformaldehyde phosphate-buffered solution (PB), cryoprotected in graded sucrose (15% (12 h) and 30% (24–48 h)) in 0.1 M phosphate buffer (pH 7.4) and frozen until processed. The stereotaxic blocks were cut in series of 45 µm thick sections in the coronal plane with a Leica CM3050 S cryostat (Leica Instruments). Sections were mounted on gelatinized slides, air dried, stained with Cresyl Violet for Nissl substance, dehydrated in a series of graded ethanol, cleared in xylene and coverslipped with Permount mounting medium. All photomicrographs were captured with a Leica DMR-E photomicroscope equipped with a digital video camera system and the Pictureframe software (MicroBrightField, Williston, VT, USA).

### Cortical microgyrus volume estimations

The brains of P120 lesion hyperthermic seizure males (LHSM, N = 5) and lesion hyperthermic seizure females (LHSF, n = 5) were cut and mounted as described above. All coronal brain sections were analyzed with Leica DMR E microscope computer assisted with the Stereo Investigator 8.0 software ((MicroBrightField, Williston, VT, USA). To estimate volumes of each lesion region, we used a systematic random sampling design [22], [23], [24], [25], [26], [27]. Sampling was done in the sensory-motor neocortex in the right hemisphere of the brain and started randomly from the most rostral section wherein the cortical lesion first appeared and finished where it ended. Subsequent systematically sampled sections were evenly spaced at 225 µm (45 µm thick, 1 every 5 sections) and a mean number of 11,16 sections stained with cresyl violet were analyzed per lesion site. Thus, an antero-posterior interval corresponding to levels between ∼2.0 and 4,5 mm posterior to bregma in the rat stereotaxic brain atlas of Paxinos and Watson (2005) was studied. The cortical lesion volumes were estimated using Cavalieri principle by point-counting [22]. Using Cavalieri method, volumes **(V) = t×ssf^−1^×ap×ΣP**; where, t: mean section thickness, ssf: is the section sampling fraction (the fraction of sections used), ΣP: sum of upper right corner points of counting frames that lie within the region of interest, and ap is the area in x-y coordinates. The volumes of the focal mycrogyrus (i.e. folding of cortical layer I within cortex) and total area of altered neocortex (i.e. all cortical tissue with loss of normal cortical lamination) were calculated from the number of points within the respective region and the distance between systematically sampled sections. Hence, for all rats, each lesion site was optically delineated at 10× with a matrix of points and the interpoint distance at tissue level was 60 µm (**see** **Figure 1 B and C** ). The precision of the sampling was assessed by the Gundersen coefficient of error (CE). This CE represents the precision in the estimation of the population size [23]. CEs were calculated for mean volumes (V) to assess the reliability of measurements. CE for the different measurements was calculated by taking the square root of the mean CE^2^. A CE of 10% (0.1) or less generally indicates adequate stereological sampling parameters [25]. Since the CE represents intrinsic methodological uncertainty, its contribution to observed variation (CV) should be less than its contribution to biological variation (BCV). The coefficient of variation (CV = SD/mean) was calculated for the volumes. The ratio BCV^2^/CV^2^, where BCV^2^ = CV^2^−mean CE^2^ and CV^2^ = BCV^2^+CE^2^, was used to determine the validity of sampling parameters. A ratio BCV^2^/CV^2^ of more than 50% (0.5) indicates acceptable stereological variability [25], [27].

### Terminal plasma costicosterone level measurements at P1 and P10

In all groups, a subgroup of pups had their plasma corticosterone levels (PCL) measured. In order to know if there were any differences between genders at baseline, we compared PCL changes at P1 or P10, before and two hours after cortical lesion or HS respectively. Baseline values were obtained before the freeze-lesion surgery at P1 via a blood sample (between 20 and 30 µL) through a jugular vein puncture and collected with a capillary tube. Two hours after the surgery, the pups were quickly beheaded and a second PCL sample was taken. Pups of the control group were separated from the dam for the same amount of time as their siblings (less than 2 min). Similar procedures were repeated before and after HS at P10 with the first blood sample collected in a capillary from the saphenous vein. The control groups, who did not undergo the lesion or HS procedures, consisted of pups that were separated from their mother for a similar period of time and sampled by the same method using a two-hour interval between measurements. Blood samples were centrifuged immediately after collection. Plasma was separated and kept in an eppendorf tube at −80°C until further processing. Corticosterone levels were measured directly in the plasma with a commercially available kit (Corticosterone ^125^I RIA Kit, Medicorp inc., Montreal, Canada). This assay enables us to measure reliably corticosterone levels greater than 0.022 nmol/ml.

### Data and Statistical analysis

All quantitative data and the statistical significance of differences between the mean values sampled within the different experimental groups were tested by a one way analysis of variance (ANOVA) or Student t-tests for independent samples. However, since data were not always normally distributed in all groups and conditions, some were compared (e.g. PCL changes at P1 or P10) with a non-parametric Kruskal-Wallis or Mann-Whitney tests. This analysis was followed by Dunn's post-hoc test when applicable. All statistical analyses were carried out with SigmaStat 3.5 for Windows with a significance level of p<0.05. All data are presented as means ± standard error of the mean (SEM).

## Results

### Increased susceptibility to hyperthermia-induced generalized convulsion (GC) in lesioned P10 pups is not influenced by gender

**Figure 2:**
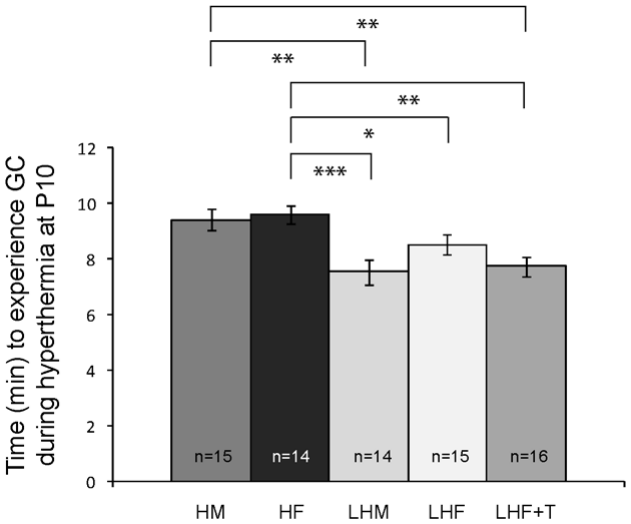
Latencies to behavioral generalized convulsion (GC) during hyperthermia at P10. Histograms showing that in the lesioned pups latency for GC is significantly shorter than in controls. This decrease is independent of gender and testosterone treatment (* = p<0.05, ** = p<0.01, *** = p<0.001).

All pups exposed to hyperthermia developed seizure-related behaviors. As previously described [3], [8], [20], arrest of movement (freezing) associated with head nodding occurred with the rise in core body temperature, followed by jaw myoclonus and a generalized convulsion characterized by tonic–clonic movements of the four limbs and loss of the righting reflex were observed. We calculated the time (in minutes) needed to experience generalized convulsions during hyperthermia at P10 in five groups of animals: Naïve males (NM, N = 15), Lesioned males (LM, N = 14), Naïve females (NF, N = 14), Lesioned females (LF, N = 15) and Lesioned females with perinatal testosterone (LF+T, N = 16). Using a one-way ANOVA test to compare the multiple groups we found several differences between controls and lesioned animals (F (4,70) = 5.737, P<0.001) (**see** **Figure 2**). With regard to the progression of seizures in controls NM vs NF the GC latencies from the onset of hyperthermia were not significantly different from each other (9.41±0.48 min vs 9.61±0.37 min, P = 0.730). Across groups (i.e. controls versus lesioned animals), GCs was significantly lower in lesioned pups both for males (7.55±0.46 min, P = 0.001) and females (7.74±0.33 min, P<0.05). LM show no distinctions with LF (P = 0.093). Finally, the latency for the lesioned androgenized females (8.51±0.45 min) is also not dissimilar from the LM (P = 0.724) and LF (P = 0.155) groups but significantly different from both the NM (P = 0.003) and NF (P = 0.001) rats. This suggests that regardless of gender, the cortical dysplasia decreases the latency to experience GC in our model. After the GC pups were removed from hyperthermia and as previously found, all LHS pups displayed persistent seizure manifestations such as myoclonic jerks and head bobbing associated with poor responsiveness, that last more than 30 minutes prior to recovery independently of genders and testosterone treatment consistent with the diagnosis of febrile status epilepticus.

### Video-EEG monitoring shows that females do not develop SRS whereas males and androgenized female rats do

#### Group 1: Males vs females

Here again, five experimental groups were tested, Naïve males (NM, N = 4), Lesioned males (LM, N = 4), HS males (HSM, N = 4), Lesioned HS males (LHSM, N = 8), Lesioned HS females (LHSF, N = 8) (**Table 1**). Behavioral and electrographic spontaneous recurrent seizures were observed only in LHSM (n = 8/8) and not in the other groups including the LHSF (n = 0/8). Baseline rhythm in all groups was a mixture of low-amplitude (<100 µV) theta and alpha waves in the 4–12 Hz range. Typical seizures are characterized by high amplitude oscillations equivalent to two to ten-fold increase of baseline rhythm amplitude with rhythmic sharp theta activity in the 5–7 Hz range generally started by an episode of slow delta waves (1–3 Hz) intermingled with polyspikes as described previously in Gibbs et al., 2011.

#### Group 2: Effects of perinatal androgenization of females with testosterone

**Figure 3:**
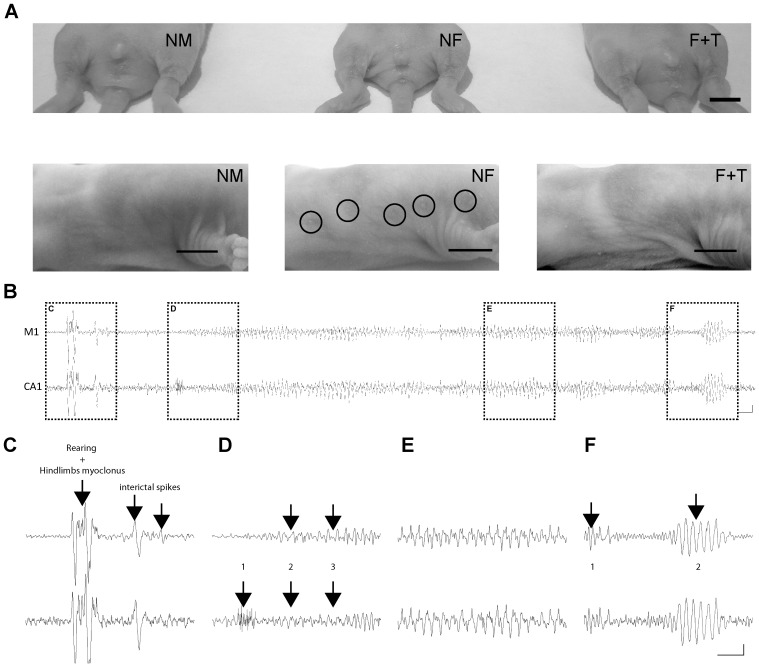
Effect of testosterone on lesioned females. **A**) Photographs showing the effects of testosterone treatment on female anatomy at P10. Male rat (NM, left), female rat (NF, middle) and testosterone injected female (F+T, right) are shown. Testosterone exposure during the fetal and perinatal periods altered the development of secondary sex characteristics in female rat (F+T) pups such as the presence of a longer anogenital distance (top) and the lack of breast (bottom). **B**) EEG in an adult LHSF+T rat showing an example of an electrographic seizure. Seizure activity recorded over a 60-second period from the right primary motor cortex (M1, top trace) and hippocampus (CA1, bottom trace) ipsilateral to the lesion. **C**) Pre-seizure baseline rhythm was composed of irregular, low amplitude, 6 to 12 Hz frequency activity. In this case, the first behavioral manifestation was characterized by abrupt rearing and hind limb myoclonus associated with the slow wave polyspiking pattern shown by the first arrow. Interictal epileptic spike or spike and wave activity was also recorded frequently throughout this period (arrows 2 and 3). **D**) Just before the seizure, the epileptic discharges were intermingled with a burst of hippocampal fast activity (1), followed by a slow rhythmic activity at a frequency of 1 to 3 Hz (2) progressing into 6 to 8 Hz rhythmic activity (3). **E**) During the seizure the EEG trace showed a prolongation of the rhythmic activity characterized by high amplitude oscillations equivalent to two to ten-fold of baseline rhythm at a lower frequency range (5–7 Hz range). **F**) The end of the seizure (1) is marked by low amplitude fast activity intermingled with periodic epileptic discharges before a progressive return to theta activity (2). The clinical manifestations included freezing followed by myoclonus, and abrupt rearing and falling. Acquisition frequency was 200 Hz, and filtering at 1–35 Hz. Horizontal bar = 1 second; vertical bar = 500 µV.

To confirm the presence of a gender-based sensitivity in our model we also studied a sixth group of pups constituted of females that received daily testosterone injections subcutaneously during early-life from E16 until P9 (LHSF+T group). The effect of treatment was confirmed at P9–P10 *in vivo* when treated females showed loss of female secondary sexual traits: specifically, the presence of a longer anogenital distance and the lack of breast development (**Figure 3A**). Contrary to the LSHF, 100% of testosterone-treated females showed interictal discharges and SRS (n = 4/4) in adulthood, which are very similar to those presently and previously found in LHSM [3], [4], [5] (**Figure 3B**).

The SRS were characterized in both groups LHSM+LHSF+T by prolonged staring episodes, head bobbing movements (Racine stage 2) and abrupt rearing with severe bilateral forelimb clonus (Racine stages 3/4). Seizures had a mean duration of 60.71±9.54 seconds and occurred between one and eight times per animal with an average of 4 episodes per 30 days for both groups. Both frequency and duration of SRS are similar to what is observed clinically. Therefore, these results suggest that, in our dual pathology MTLE model, only male and androgenized female develop SRS later in life as they express similar seizing patterns.

### No gender differences in cortical lesion volumes in P120 LHS rats

To test if differences between male and female adults could be explained by differences in volumes of cortical lesions, we determined the volume of the lesioned area by stereological analysis from systematic serial brain sections using point counting and the unbiased Cavalieri's rule (see methods above). The estimation of cortical volumes for the total lesioned site and mycrogyrus only produced a mean BCV^2^/CV^2^ ratio of 0.91 and 0.97 respectively in the LHMS group. In LHFS animals these ratios were similar with values of 0.96 and 0.98 correspondingly. This indicates stereological sampling parameters and that more than 90% of the observed variation reflects biological and not methodological differences. The average Gundersen coefficient of errors (CE's) for volumes was well below 0.10 (LHSM, CE = 0.038 and LHSF, CE = 0.047) indicating a good accuracy for these sampling parameters. Student t-test analysis showed no significant differences in total cortical volumes of lesion site (i.e. 2.09±0.21 vs 2.01±0.26 mm^3^, P = 0.763) and mycrogyrus alone (i.e. 0.41±0.06 vs 0.39±0.09 mm^3^, P = 0.573) between the two dual pathology groups of different gender (**see** **Figure 1 A and B** ). Altogether this suggests that the cortical lesion volumes are not affected by gender.

### Significant rise of plasma corticosterone at P1 correlates with long-term outcome

**Figure 4:**
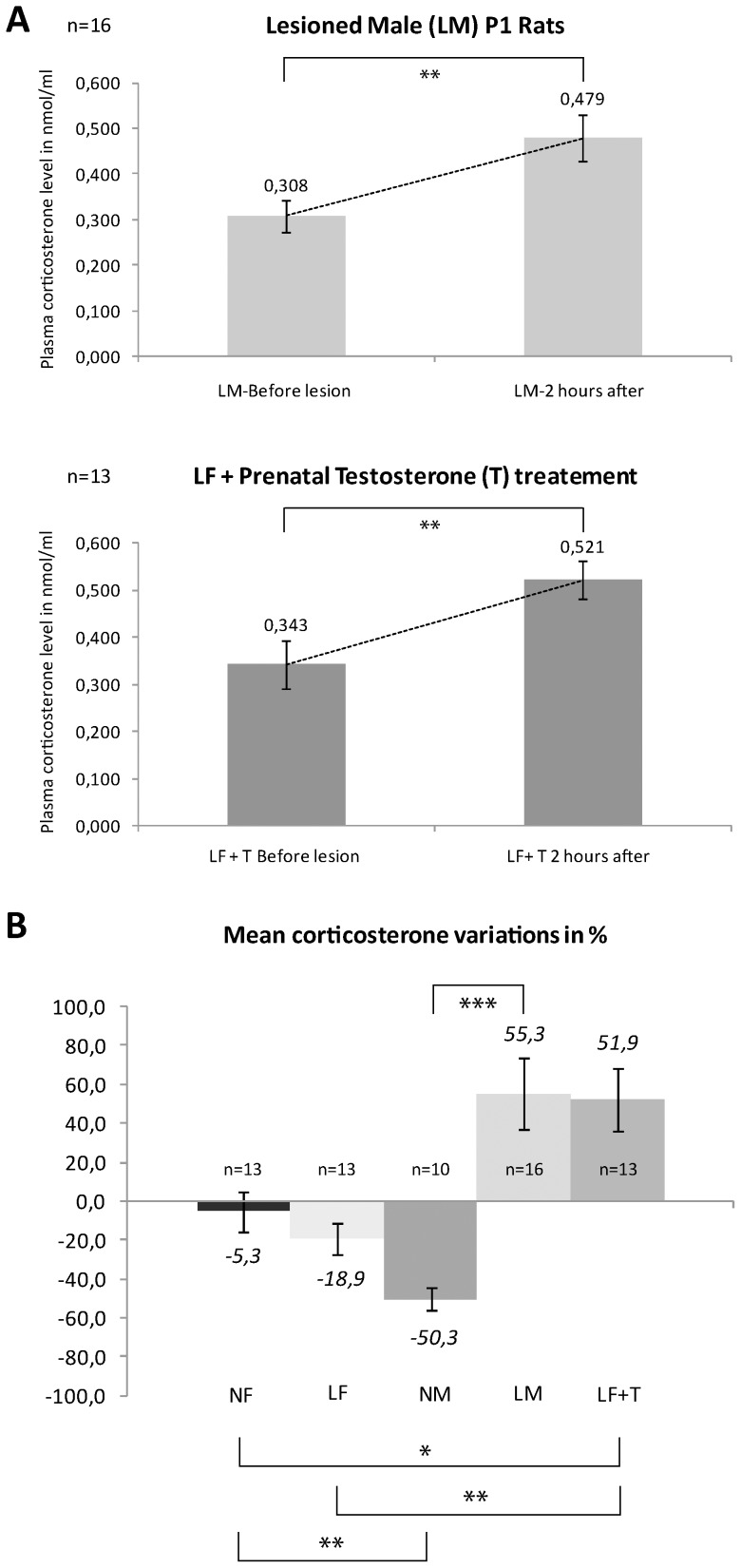
Plasma corticosterone level (PCL) changes following treatments. **A**) Histrograms showing changes in PCL before and after the cortical lesion in nmol/ml at P1 in males (top) and in females, bottom). **B**) Mean PCL variations for all the experimental groups in percentages. Note that the LM and LF+T groups were the only ones that showed an increase of corticosterone levels following cortical dysplasia induction at this age, whereas the others showed diminution. These increases were statistically different from their controls, notably LM compared to NM (*** p<0.001), LF+T compared to NF (* p<0.05) and to LF (** p<0.01).

At P1 five groups were established: Naïve males (NM, N = 10), Lesioned males (LM, N = 16), Naïve females (NF, N = 13), Lesioned females (LF, N = 13) and Lesioned females with perinatal testosterone (LF+T, N = 13). An important inter-individual variability was observed in the plasma corticosterone levels (PCL) in all pups. As all our animals had PCL taken before and after the freeze lesion, we decided to compare in each pup, the variation in PCL as expressed in percentage of increase or decrease from baseline. First, we observed no significant difference in the PCL between genders at baseline, prior to the cortical dysplasia induction at P1 (F (1,49) = 0.734, p = 0.401). However, males and females demonstrated significant differences in their PCL response at P1, 2 hours after the lesion. The only groups that showed a rise in their PCL in the present paradigm were the LM and LF+T animals. On average, the LM increased by 55%, which was significantly different from the naïve males (NM) (p<0.001); and the LF+T increased by 52% which was significantly different from the naïve females (NF) (p = 0.01) but also from the lesioned females (LF) (p = 0.009) (**Figure 4 A, B**). Naïve groups of both genders showed a reduction in PCL, after a separation time corresponding to the surgery plus recovery time in the lesion group siblings with a significantly greater reduction in males as compared to females (p = 0.004). We also compared the changes in PCL before and two hours after hyperthermia at P10 between males and females but although levels were raised in all groups, that increase was not significantly different between groups (F (4,22) = 0.215, P = 0.927, data not shown). Overall our results show that, in our model, only male and testosterone-treated female animals demonstrate a strong and positive stress response after the first insult at P1. It also points out that, at this age in our rat model, stress responses translated by PCL fluctuations are more sensitive to environmental cues in young naïve males than in females.

## Discussion

The main finding in this study is that only lesioned males (LM) and androgenized lesioned female (LF+T) pups display spontaneous recurrent seizures after experiencing prolonged HS. The distinctive point about these two subgroups is the significant increase in PCL after the induction of a focal cortical lesion 24 h after birth whereas the normal females undergoing similar brain insults do not develop epilepsy or changes in PCL.

### The cortical lesion volumes in adulthood are independent of gender

Neonatal freeze lesions to the cortical plate result in focal malformations of the cerebral cortex that resemble a four-layered microgyrus. These malformations have been associated with local and distant changes up to 3 mm in neuronal architecture. Although the size and severity of these lesions could explain differences in outcome, by measuring the cortical lesion volumes in our LHSM and LHSF adult rats we observed no significant differences between groups. These results are quite similar to what was thoroughly and carefully studied in Wistar rats that underwent early freeze lesion by Rosen et al. in the late 1990's [14], [28], [29]. In these studies the lesion volumes were comparable to the present study and were also determined from Cavalieri's rule. Consequently, they found that the freeze injury to the cortical plate of rats induced an indistinguishable cerebrocortical microgyria phenotype between males, females and androgenized females. Altogether, this also points out that the freeze lesion methodology is rigorous and reproducible between groups [2], [4], [14], [20], [29], [30]. The current data also emphasizes that HS in pups with a localized cortical lesion results in lower generalized convulsion (GC) threshold and lead to non convulsive status epilepticus. However, as the cortical lesion is similar between genders and that the animals that experience only the lesion do not develop SRS in adulthood, both insults (LHS) and possibly other systemic molecular factors or hormones [2], [4], [31], [32] may be required to facilitate circuit reorganization and epileptogenesis.

### A greater vulnerability to GC during hyperthermia in both male and female lesioned pups

Across genders the GC latencies were very similar but significantly decreased in all lesioned pups. This result is analogous to what we have found previously in LHSM versus NHSM rats [3], [20]. Previous EEG recordings have shown a mean duration of hyperthermic SE of 34.7±7.4 min in LHSM pups and 25.4±4.9 in HSM controls (Gibbs et al., 2008). Likewise, even though, we did not record ictal EEGs at this age, we observed that all LHS animals (i.e. LHSM, LHSF and LHSF+T) only recovered their righting reflex after more than 30 minutes.

In unlesioned rats, it was formerly demonstrated by Baram et al in 1997, that close to 33% of male rats that underwent hyperthermia for a long period lasting almost 30 minutes (≈20 minutes post generalized convulsion) became epileptic [8], [33]. The same group further reported that when unlesioned rats are subjected to even longer prolonged hyperthermic seizures (i.e. over 60 minutes), SRS are much more longer and severe although occurring only in a similar proportion of animals [34]. At odds with these earlier studies, we did not observe SRS during the prolonged video-EEG monitoring of unlesioned male rats following hyperthermic seizures [2], [4], [5], [7], [20], [33]. Whereas seizure duration is approximately the same in both protocols, it should be noted that the exposure to hyperthermia was shorter in our experiment, this could explain the observed differences in outcomes [9]. Our model is indeed different and uses a shorter standardized exposure to hyperthermia for all animals since we take them out of the heating cage as soon as GC appears. Overall, these data suggest that although normal pups may have the potential to develop SRS following a prolonged hyperthermic SE, the epileptogenic effect of such seizures is enhanced by a pre-existing brain anomaly.

Since both genders react in a similar fashion during and after hyperthermia-induced GC, we tested the hypothesis of a specific implication of early-life stressors (e.g. rise in glucocorticoids) and testosterone on increasing stress-induced limbic modifications and ensuing epileptogenesis in this specific dual pathology rat model of MTLE. This hypothesis is supported by the finding of increased levels of plasma corticosterone only in LM and LF+T rats at P1 that once subjected to hyperthermic seizures at P10 are developping SRS later in life whereas all the other groups with low corticosterone levels at the time of the cortical lesion don't.

### The role of early-life stress in promoting epileptogenesis

Many epilepsy patients and clinicians report that stress can provoke and/or aggravate epilepsy [1]. However, little is known about the physiological consequences of early-life stress on epileptogenesis [17], [35]. Both procedures in our model are considered stressors and are associated with variations in corticosterone levels. However, only the initial insult at P1 (freeze lesion) displays sexual dimorphism in corticosterone response. Hyperthermic seizures at P10 are also followed by elevated PCL compared to baseline, suggesting that prolonged seizures are a significant insult, but at this age the rise in PCL affects all experimental groups without any gender differences. Together, these results may highlight the presence of a critical period for gender-based differences in stress responses to specific early life insults that occur around birth in the Sprague-Dawley rat strain at least in this experimental model.

Mechanisms that may underlie the predisposing effect of corticosterone on temporal lobe epileptogenesis involve the hippocampus and the amygdala as they contain high densities of mineralocorticoid (Type I or MR) and glucocorticoid (Type II or GR) receptors [36], [37], [38]. In many animal models, stressors or supplementation of corticosterone lead to morphological or functional changes in hippocampal structures that are similar to those observed in TLE patients [17], [39]. For example, administration of high doses of glucocorticoids results in hippocampal atrophy through the reduction of dendritic processes, the loss of pyramidal cells in the CA1 and CA3 regions, and inhibition of dentate granule cell neurogenesis [40]. These abnormal changes are further supported by the observations that prenatal and postnatal stress experiences generally facilitate seizure development and decrease threshold to kindled seizures in adult rats [12], [16], [17], [35], [39], [41]. This effect on seizures could certainly be mediated by corticosterone, as supplementation with the stress hormone in adult rats accelerates electrical kindling [42]. Furthermore, the facilitating effect of corticosterone on lithium pilocarpine-induced status epilepticus in P10 rats is inhibited by metyrapone, a blocker of corticosterone synthesis [43] confirming the important role of corticosterone in epileptogenesis. High corticosterone levels in early-life are thought to irreversibly impair the inhibitory control of the HPA axis. The intact hippocampus usually having an inhibitory control on the HPA axis secretion and the amygdala a driving one [15], the disinhibition of this chronic stress axis may also, in conjunction with corticosterone accumulation, produce an hypersecretion of Corticotropin-Releasing Factor (CRF) from the paraventricular nucleus, a peptide well known to have a proconvulsive effect in young rodents [44], [45].

Sexual dimorphism linked to early-life stress responses in adulthood seizure susceptibility and occurrence has been reported in only few studies of established animal models of TLE, notably the kainic acid model [46] and the kindling model [12], [16], [18], [19], [35] but with conflicting results. For example, Edward and collaborators (2002) found that early-life stress in Wistar rats can facilitate the development of kindled seizures in adult rats in a gender-dependent manner, males also being more affected. However, Salzberg et al. (2007) found that early postnatal stress, using maternal separation, confers enduring vulnerability to limbic epileptogenesis, illustrated by increased kindling rate and reduced seizure threshold, but only in stressed females. Another study from the same group reported that some male individuals could also be affected [18]. More recently, they have also shown rats exposed to maternal separation stress in early postnatal life had accelerated kindling rates in adulthood and total CA3 pyramidal cell numbers were also reduced in both gender. However, separated female rats had heightened corticosterone responses and dentate granule cells neurogenesis after kindling, whereas males had a similar but not significant trend [19]. These variable results could reflect differences in experimental paradigms used, type and timing of the insults, physiological responses to specific noxious stimuli and the genetic background of each rat strains. Overall, these experiments demonstrated that early life stress results in enduring enhancement of HPA axis responses to limbic seizures in a sex-dependent pattern. This implicates important candidate mechanisms through which early life stress may promote vulnerability to limbic epileptogenesis in rats as well as to human MTLE and its associated psychiatric disorders. Our results show that in our lesion+hyperthermia rat model of MTLE females do not develop SRS in adulthood unless they are treated with testosterone in perinatal life.

It is well known, in Sprague Dawley rat pups, that early in development hyperpolarization of GABA_A_ reversal potential (E_GABA_) appears earlier in female (∼P10) than in male (∼P14) CA1 pyramidal neurons [47], [48]. It has also been shown that prolonged maternal separation prematurely augments hyperpolarizing GABA_A_ responses in both sexes, yet through different molecular pathways. In male pups, this is affected by increase in KCC2 and less active NKCC1 chloride cotransport where as in females, this is attributed only to lower NKCC1 expression [49]. Taken as a whole these results suggest that GABA_A_ receptor signaling may be key mechanism for the age-, stress- and sex-specific sensitivity to seizures in the hippocampus as it is involved in normal brain development, early-life stress responses and possibly epileptogenesis. In the present study, male rat pups may be more sensitive to stressful environmental changes during postnatal life because their inhibitory system development is protracted compared to female siblings meaning it may maintain an excitatory GABA_A_ phenotype, for a longer time early in life. Therefore, this maturation window could perhaps serve as a sensitive period for insults in our model and better explain why the males are more sensitive to stressful postnatal insults with the development of spontaneous seizures later in life as compared to female siblings that are not affected.

### Sex hormones also influence epileptogenesis in the present dual pathology model

There are no clear data on the gender distribution of MTLE in humans, especially in patients with a past history of prolonged febrile seizures. In fact, the only epidemiological study looking at febrile seizures and prenatal stress did not find a causative effect, but authors looked at all febrile seizures most of which are probably genetically influenced, rather than specifically at prolonged febrile seizures [50]. Using video-EEG monitoring, we demonstrated that lesioned female rats are resistant to epileptogenesis in our model despite experiencing prolonged HS and that the initial insult does not lead to a significant stress response unless they are androgenized. It thus appears that more than just the cortical malformation is required to increase vulnerability to the prolonged HS and lead to epileptogenesis in female rats. The freeze-lesion model has been reported to induce changes in brain excitability, but these changes never manifest as spontaneous recurrent seizures in later life [2], [4], [14]. Similarly, HS in naïve male rats when associated with prolonged exposure to hyperthermia (>30 minutes) can lead to MTLE in 33% of adult rats [8], but when hyperthermia is brief (∼10 minutes) , MTLE is only seen when combined with a predisposing lesion [2]. We therefore believe that the observed changes in corticosterone levels, seen in males only after the insult at P1, contribute to increasing the risk of epileptogenesis. Furthermore, this hypothesis is reinforced by the observation that androgenized females have a propensity to develop a significant stress response after the cortical lesion and develop SRS following HS later in life.

Long lasting gender differences in the effects of early-life stress on the brain development have long been reported in the literature [51]. Rosen et al (1999) suggested that high levels of androgens acting in the developing male brain may render it somehow «hyper-reactive» [14]. In rats, the brain is very sensitive to the organizing effects of androgens especially testosterone during the first week of life. Male pups experience a surge of testosterone that occurs on days 18–19 of gestation and again during the first few hours after birth with a critical window from P0 to P2 [14], [52]. Emerging data show that testosterone and its active metabolites, estradiol and dihydrotestosterone, are important factors in both androgenization of the brain and the response to early life insults such as cerebral ischemia, trauma and seizures [53]. In general, clinical evidence and animal models indicate greater brain damage in newborn male compared to female following injury. Nevertheless, very few studies have compared male and female rats in seizure susceptibility using animal models of MTLE [12], [16], [54], [55]. Testosterone has been shown to contribute to the excitotoxicity of seizures in the kainic-acid rat model of MTLE [54]. Daily postnatal administration of testosterone (from P0 to P14) to castrated males or to intact female rats results in a male proconvulsant phenotype to flurothyl-induced seizures in P15 rats [56]. This could justify the use of sex steroid antagonists not only as anticonvulsants but also as antiepileptogenic agents in the developing brain [57]. Studies have shown that when testosterone is aromatized into estradiol (E_2_), it can also have proconvulsive effects in several animal models lowering seizure threshold and enhancing the rate of recurrence [58]. This molecule enhance ionotropic glutamate receptors (e.g. NMDA receptor 2B) mediated excitation in area CA1, a receptor involved in the hyperexcitability observed in our model [59], [60]. This testosterone metabolite can also inhibit GABAergic transmission by decreasing the effect of GABA at the GABA_A_ receptors of CA1 pyramidal cells [61]. It decreases the slow after hyperpolarisation mediated by Ca^2+^-dependant potassium (K) currents [62]. Nunez and Macarthy reported that using a GABA_A_ agonist can induce hippocampal cell death in the newborn rat, that these changes are more severe in males and can be accentuated by estradiol pre-treatment [63]. In general, all these phenomena can contribute to the generation of hyperexcitability and injury in the epileptic brain.

In parallel, in adults, stress can inhibit gonadotropin secretion and reproductive function. Contrasting to this decrease of bioavailable testosterone, levels of corticosterone are usually greater among individuals with acquired epilepsy compared to controls [17], [35], [53], [64]. Thus, in our model, high levels of corticosterone may decrease free testosterone, but increase E_2_ levels in an age-dependent fashion. Because androgens can modulate HPA axis function, perinatal androgen dysregulation may contribute to seizure susceptibility either directly, via actions at androgen receptors, glutamate or GABA_A_ receptors, or indirectly by altering HPA axis secretion. The present findings suggest that in the early postnatal period testosterone exposure in conjunction with an atypical activation of the HPA axis function during that same period may have important implications in establishing hippocampal excitability in these animals. The exact interplay or synergistic pathways between sexual and stress steroids on epileptogenesis remains to be elucidated at this point and further studies will be needed to gain a better understanding of the exact changes involved in these processes. However, our results clearly show that the presence of both testosterone and corticosterone promotes sensitivity to the second insult (HS) at P10 and facilitates recurrent seizures in a gender-based manner in the present rat model of MTLE.

### Conclusion

An early brain injury after birth predisposes the developing brain to the deleterious effects of HS in a gender specific manner. The combination of testosterone and its metabolites plus elevated corticosterone levels at P1 lead to a selective functional vulnerability of the hippocampus which is a common loci for FS, MTLE, stress biology alterations, and androgen-receptivity. These early-life changes combine to increase the vulnerability of the hippocampus to prolonged HS, leading to MTLE later in life. This could eventually contribute to the general vulnerability of males to early-life insults. We anticipate that this model will permit us to gain more mechanistic insights into the relation between early-life stress, prolonged febrile seizures and epilepsy. Furthermore, our findings support the strategy of modulating sexual or stress steroids as preventative therapy in the developing brain.
